# Evaluation and promotion strategy of resilience of urban water supply system under flood and drought disasters

**DOI:** 10.1038/s41598-022-11436-w

**Published:** 2022-05-06

**Authors:** Zhijie Li, Hui Zhao, Jinning Liu, Jingqi Zhang, Zhiguo Shao

**Affiliations:** 1 School of Management Engineering, Qingdao University of Technology, Qingdao, 266520 China; 2 Tongji University Sustainable Development and New-Type Urbanization Think-Tank, Shanghai, 200092 China

**Keywords:** Climate sciences, Environmental social sciences, Risk factors, Engineering

## Abstract

With global climate change and the rapid urbanization, urban flood and drought disasters are frequent and urban water supply systems are facing a sea of serious challenges. It is crucial to assess the resilience of urban water supply systems and develop corresponding disaster mitigation and improvement strategies. Urban water supply systems include many subsystems, but existing researches generally focus on a single subsystem. Therefore, this paper proposes a correlation analysis method and a factor analysis method for the resilience evaluation index system of urban water supply systems by combining each subsystem and applying grey system theory. The method can reflect the four dimensions of the water supply process (water source, water plant, supply and distribution network and users) and the five dimensions of the urban management system (society, natural environment, economy, physics and organization). Taking Qingdao as an example, a multi-level integrated evaluation model based on a cloud model is applied to simulate and analyze the resilience of Qingdao's water supply system. As a result, decision support is provided for planning and building resilience systems for urban water systems in the short and long term, based on four main factors.

## Introduction

In the context of global warming and rapid urbanization, the probability of future extreme weather events (such as extreme temperature and abnormal rainfall) is likely to increase dramatically. These hazard events greatly exacerbate the impact on the safety of urban water supply facilities^1^ and the safety of water supply system has become more complex and uncertain than ever. Therefore, the water sector should assess the safety and resilience of urban water supply systems under extreme events^2–5^. Urban water system is a general term for water-related matters such as flood control, water source development, water supply, water transmission, water use, drainage, wastewater treatment and reuse, inter-regional water transfer and etc. It is used for the development, utilization, treatment and distribution, of water resources in urbanized areas, as well as for conservation and protection^6^. Urban water supply systems are usually defined as water supply systems that take water from a water source and transport it through a transmission pipeline to a waterworks for water quality treatment. The treated water is pressurized and transmitted to consumers through the distribution network. It consists of the water source, the water plant, the power supply and distribution network and the consumers. Failure of the urban water supply system can not only affect the normal production and life of the city, but can also lead to the collapse of the whole city^7^. Therefore, as the lifeline system of a city, how to ensure the safety of urban water supply under extreme weather events has become an urgent issue to be addressed.

At present, relevant studies are mainly concentrated in the fields of environmental science and ecology, water resources, engineering and technology, with fewer urban research areas. Relevant domestic research started late, most of the literature is at the preliminary theoretical stage, and the number of studies is small. A single evaluation method is mainly used, with rigid evaluation indicators and a single dimension of concern. The evaluation model is difficult to apply in practice and there are few empirical studies. Some studies have evaluated the resilience of the water source system and the supply and distribution network system of urban water supply systems, but the researches tend to focus on only one subsystem of the water supply system. There are also few reports on resilience assessments of water supply systems under flooding. Therefore, on the basis of summarizing the existing research results, this paper constructs a multi-dimensional evaluation indicator system for the resilience of the water supply system based on correlation analysis and factor analysis from the perspective of the resilience of the water supply system. A multi-level comprehensive evaluation model based on a cloud model was established to simulate and analyze the resilience of the water supply system in Qingdao. The main factors affecting the resilience of the water supply system were identified, and in response to these main factors, combining structural and non-structural measures, this paper proposes strategies for improving the resilience of the urban water supply system, with a view to providing theoretical support for the short- and medium-term planning of the water supply system.

## Literature review

### Literature visualization analysis

**Figure 1:**
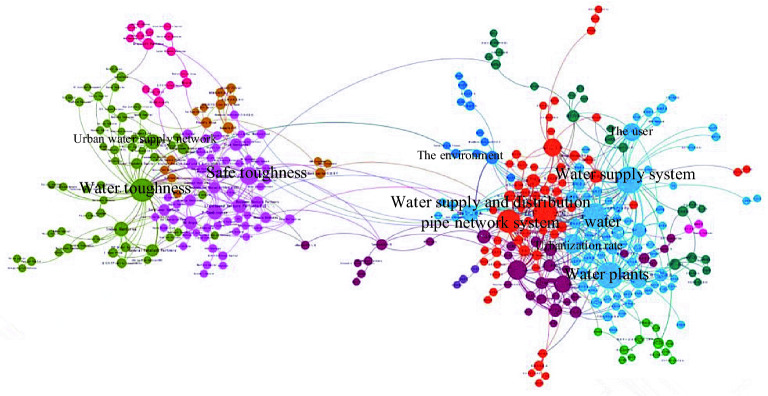
Visual analysis diagram of literature related to urban water supply systems (the figure shows a visual analysis of literature in the last 10 years, with foreign countries on the left and China on the right).

CiteSpace software (https://citespace.podia.com/download, Version 5.8. R3) was used to visualize and analyze the literature related to urban water supply system over the past 10 years, and the results are shown in Fig. 1. It was found that urban water supply systems usually include subsystems such as water sources, water plants, supply and distribution pipeline network and consumers. However, there has been more researches on the evaluation of the resilience of water source systems and supply and distribution pipeline network systems in urban water supply system, and a series of research results have been achieved, enriching the connotation of the resilience of supply and distribution systems^8^. But the research seems to focus on a particular subsystem of the water supply system. There are few studies on the construction of a resilience indicator system and the assessment of the resilience for the whole water supply system under flood and drought disasters.

### Research status

Fiering^9^ and Hashimoto et al.^10^ studied the water source resilience of urban water supply systems earlier and used a mathematical model to evaluate the exact values. The model is easy to understand but difficult or slow to apply in practical cases. Tanner et al.^11^ assessed the resilience of water sources from a social policy perspective, but the indicators could not be quantified and did not consider the resilience of urban ecosystems. Qiao et al.^12^ investigated how to protect water supply network nodes and maximize the resilience of water supply networks within a limited capital budget. Gay and Sinha^13^ considered the resilience of an urban water supply network system as the probability of recovering from a disaster within an acceptable time and cost, and estimated the resilience of water supply network through a stochastic simulation method. Arka and John^14^ proposed a new water system resilience index based on the water system network topology by combining six network attributes of the urban water system and assigning weights to the six network attributes through an analytical hierarchy process. Zhao et al.^15^ studied the recovery strategy of water supply systems under sudden water pollution events and established the recovery strategy optimization and selection model. The research showed that reasonable strategy optimization and selection can effectively shorten the emergency recovery time and improve the recovery capacity of water supply systems. Based on the concept and connotation of urban flooding resilience, Xu et al.^16^ established a grey box model and constructed an evaluation system of urban flooding resilience including three dimensions of resistance, resilience and adaptability with the help of principal component analysis, and evaluated the flooding resilience of 238 prefecture-level and above cities in China. Based on the concept of system elasticity and DO-E2S2 system analysis framework (the so-called DO-E2S2 system analysis framework refers to the six system dimensions (population, organization, economy, environment, infrastructure and socio-cultural system) closely related to the water supply system, as well as the system comprehensive evaluation index system reflecting the resilience of the water supply system, such as absorption capacity, adaptability and recovery capacity), Liu J and Huang W^17^ established an urban water supply system elasticity evaluation index system reflecting three dimensions of system absorption capacity, adaptive capacity and recovery capacity, and used GIS technology to visually evaluate the system elasticity of a water supply region in Shanghai under a salt tide scenario, giving the evaluation results of system resilience and early warning zones based on continuous improvement of system quality. Yu et al.^18^ described the concept, development and application of elasticity in the field of urban water systems, reviewed the evaluation methods and elasticity strategies of water system resilience, and looked forward to the four development trends of water system elasticity research according to the development background of existing research. Li et al.^19^ combined previous research results on the water supply systems and proposed that the meaning of seismic toughness of water supply systems should include seismic safety and post-earthquake recovery capacity. The evaluation method also gave an evaluation model framework that integrated multiple evaluation indexes from the perspective of seismic safety and post-earthquake recovery capacity, and pointed out that the behavioral criteria for seismic resilience evaluation of water supply systems should match the requirements of the current seismic codes and setting standards.

Although the above research methods have achieved a series of results that can be used to enrich the connotation of water supply system toughness, on the one hand, the current resilience evaluation mostly adopts the comprehensive evaluation index method and the entropy-weight-based clustering method, and there are few evaluation methods. On the other hand, in spite of the fact that the comprehensive evaluation indicators can reflect the development trend of toughness well, the classification accuracy of the evaluation results is closely related to the number of original samples and data. In order to obtain a reasonable evaluation grade, a large number of samples from different regions need to be collected. Therefore, this method shows certain limitations when only a certain region is evaluated.

## Theoretical basis and research framework

### Theoretical basis

#### Urban water supply systems

An urban water supply system is usually a system where water is taken from a water source and transported through a transmission pipeline to a waterworks for water quality treatment. The treated water is pressurized and transmitted to consumers through the distribution network. It consists of water source, the water plants, the power supply and distribution network and the consumer. On this basis, this paper also takes full account of other management systems that are closely related to urban water supply system, such as urban society, the natural environment, the economy, the physical attributes, organizational systems, and flooding and drought disasters, etc.

**Figure 2:**
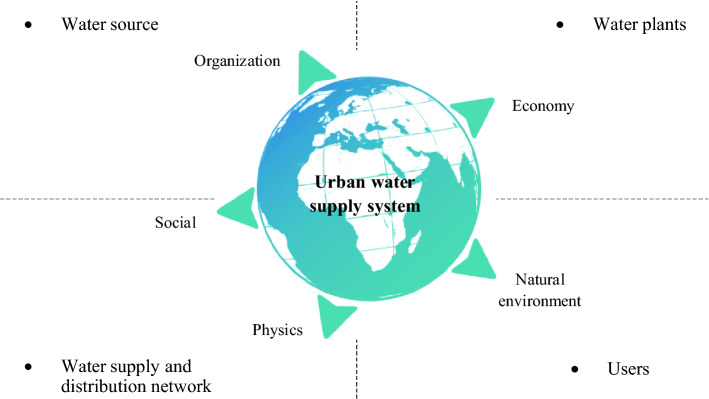
Urban water supply systems (the figure shows four dimensions of whole-process water supply system and five dimensions of urban management system).

Therefore, the urban water supply system defined in this study includes not only the four water supply system dimensions of water source, water plant, supply and distribution network and consumers. It also covers the five urban management system dimensions of society, natural environment, economy, physics and organization, as shown in Fig. 2.

#### Resilience concept

In recent years, the research on the resilience of urban water supply system has received increasing attention from scholars. The so-called resilience of water supply systems refers to the ability of water supply systems to resist disasters, reduce disaster losses, allocate resources rationally and recover quickly. The English word for “resilience” is derived from the Latin word “resilio”^20^. At present, there are three main ways to translate “resilience” in China, resilience, elasticity and toughness. Wang et al.^21^ showed through their research that the unified translation of “resilience” is closest to the mainstream understanding of its academic connotation. In contrast to toughness and elasticity, the term resilience encompasses the sublimation of the idea that systems return to a state of equilibrium after disturbance. It suggests the sense that the human world, as a social-ecological system, should not return to square one after a catastrophe, but rather become stronger, more prosperous and more resilient after suffering a disaster. Holling, a professor of ecology at the University of Florida, introduced the concept of “ecosystem resilience” in his book *Resilience and Stability of Ecological Systems* in 1973^22^. He is the first to import the concept of resilience into ecology. He defined the concept of resilience as the ability of a system to maintain some original state after a disturbance. Later, some scholars defined the concept of resilience as “engineering toughness” from the perspective of the speed of recovery of a system after an interference^4^. Since then, the concept of resilience has been widely used in many fields, such as engineering systems, socio-economic systems, water ecosystems and urban infrastructure systems.

Therefore, this paper argues that the toughness capacity of the supply and distribution system refers to the ability of the supply and distribution system to withstand disasters, reduce disaster losses, allocate resources rationally and quickly restore normal water supply after a disaster.

#### Cloud model concept

Academician Li D^23^ first defined the concept of a cloud model, which is a mathematical model with uncertainty in which qualitative descriptions and quantitative concepts are transformed into each other. The existing qualitative evaluation mainly suffers from subjectivity and arbitrariness. The advantage of the cloud model is that it can overcome these disadvantages and effectively evaluate the evaluation object.

Let *u* be a domain of quantities represented by values and *C* be a qualitative concept on *U*. If a quantity *x* ∈ *U* is a random realization of a qualitative concept *C*, *x* is deterministic with respect to C and *u*(*x*) ∈ [0,1] is a random number with a stable tend:

1 $$u:U \to \left[ {0,1} \right],\forall x \in U,x \in u\left( x \right)$$

Then the distribution of *x* in the domain *U* is called the cloud model and is determined as *C*(*x*). Each *x* is referred to as a cloud droplet.

**Figure 3:**
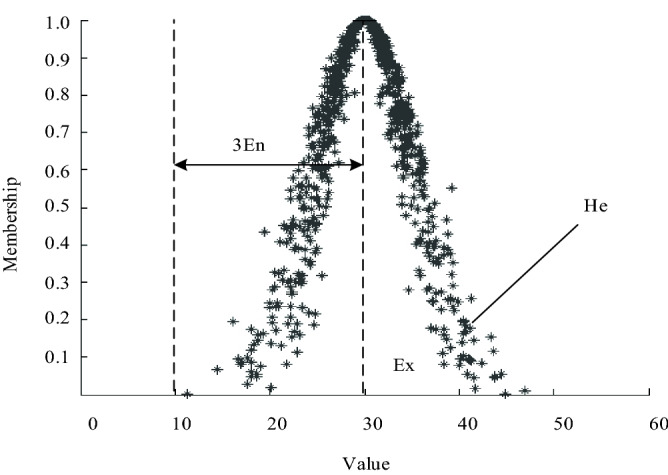
Schematic illustration of the digital features of the cloud.

When representing a concept as a whole, three numerical features are used to realize it, namely the expectation *Ex*, the entropy *En* and the hyper metropy *He*, as shown in Fig. 3.

### Research framework

**Figure 4:**
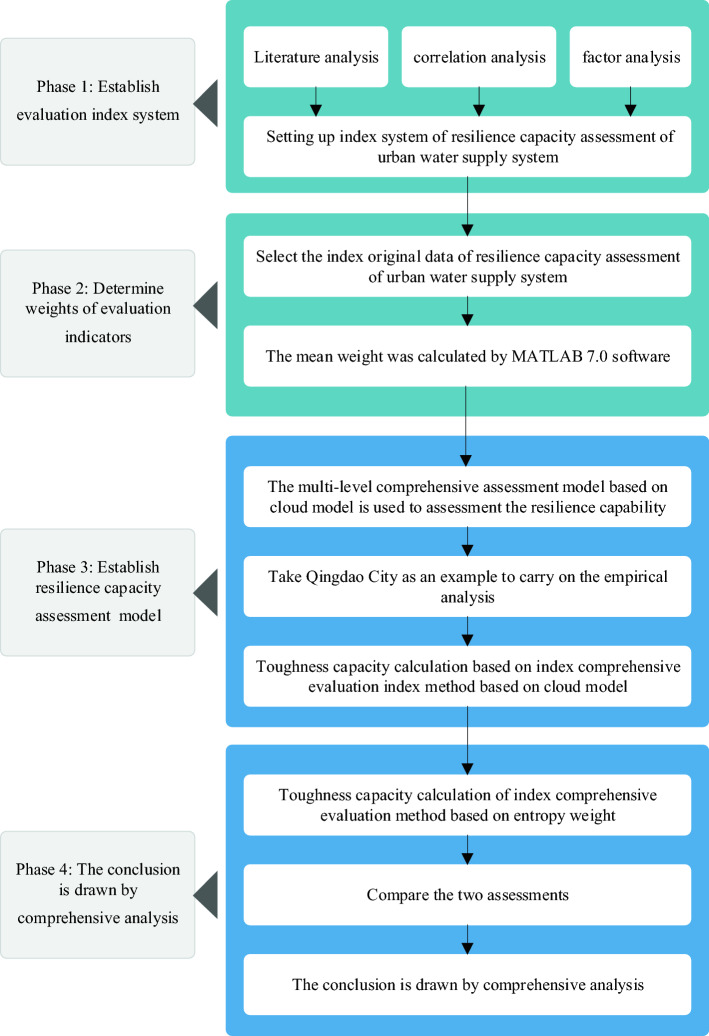
Research framework (from top to bottom, the figure shows the logical process and methods of toughness evaluation of urban water supply system in four parts).

This study constructs a resilience evaluation index system for urban water supply systems from the two dimensions, organization system and flood management system. A multi-level comprehensive evaluation model based on the cloud model is used to evaluate the resilience of the urban water supply system. Based on the evaluation results, the main influencing factors of toughness of water supply system are analyzed and strategies for improving the resilience of the water supply system are given. Decision-making support is provided for the short-term and long-term planning and construction of the resilience system of urban water supply systems. The research framework of this paper is shown in Fig. 4.

## Index system of resilience capacity assessment of urban water supply system

In this paper, the resilience assessment framework for urban water supply systems constructed by Balaei^24^ and Lukuba et al.^25^ is improved and applied to the resilience capacity assessment of urban water supply systems in China. On the basis of considering the whole process management of urban water supply systems, the minimum necessary associated urban management system closely related to the urban water supply system is fully considered (as shown in Fig. 2). The urban water supply system resilience capacity evaluation index system is further refined.

### Principles and methods of index selection

**Figure 5:**
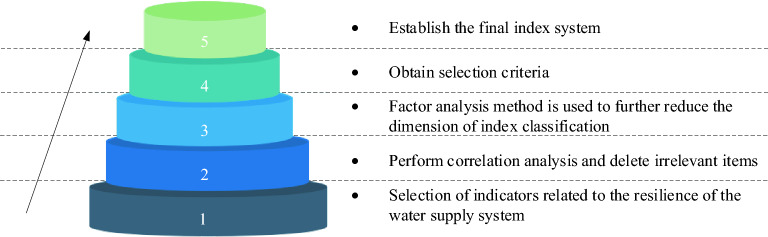
Selection model of toughness index of urban water supply system (according to the arrow direction, from bottom to top, show the establishment process of toughness index selection model of the urban water supply system).

The resilience evaluation indicators for urban water supply systems were selected based on the principles of representativeness, feasibility, non-repetitiveness and conformity to the meaning of resilience^26^. In accordance with the above principles, an open "cylinder index selection model" was established, as shown in Fig. 5. The resilience index of the urban water supply system was selected through a hierarchical filtering process.

#### Basic data preparation: audition

Following the above selection model and selection principles for toughness indicators, all indicators related to the toughness of the water supply system, including water source, water supply and water consumption, can be subsumed into the alternative index system (see Appendix 1 for indicator sources and data sources).

#### Index correlation analysis

In order to ensure the scientific, rational and representative nature of the selected indicators, sample data from 31 provinces and provincial capitals directly under the central government from 2011 to 2020 were selected, and 48 indicators were chosen based on feasibility and continuity. According to the research background of the problem, and considering that the threat of flood and drought disasters also affects the toughness of the water supply systems, two proxy indicators for flood and drought disasters were added to the above indicators, the end-of-year storage capacity of reservoir and the number of people affected by flood and drought. Indicators and data sources, National Water Resources Statistical Annual Report, China Urban Construction Statistics Yearbook, National Bureau of statistics, provincial water resources bulletin, relevant references, etc.

The Pearson correlation coefficients^7^ between each index and per capita water resources, comprehensive production capacity of water supply, urban residential water consumption, industrial enterprises' water consumption, and the number of people affected by flood and drought were calculated to obtain the correlation level and significance test values. For rigorous screening, the significance level test threshold was set at 0.01. After analyzing and removing six less relevant indicators, including population density, number of people aged 6 and above with tertiary education, leakage rate of the pipeline network, water supply module, loss of water facilities due to disasters, and regional GDP index, 42 indicators related to the resilience of urban water supply systems were finally obtained (five categories of indicators overlapped, and the overlapping indicators were calculated only once).

#### Index selection is based on principal component factor analysis

The two indicators of total water supply and total water consumption were not suitable for factor analysis as they were highly correlated. After general consideration, factor analysis was carried out on the remaining 41 indicators excluding the total water consumption indicators. KMO and Bartlett's tests should be carried out prior to factor analysis of the data and the final test results are shown in Table 1.

**Table 1:** KMO and Bartlett test results.

Sampling the Kaiser–Meyer–Olkin measure of adequacy		0.822
Bartlett’s test for sphericity	Approximate chi-square	11,443.479
	*df*	820
	*Sig*	0.000

KMO value was 0.822 and the corresponding *Sig* value was 0.000, indicating that the raw data used for the indicators in this paper were suitable for factor analysis. Factor analysis was performed on the data to yield the total variance explained, as shown in Table 2. The extraction method was the principal component analysis^7^.

**Table 2:** Total variance explained.

Composition	Initial eigenvalue			Sum of squares of the extraction load			Sum of the squares of the rotating loads		
	Characteristic root	Variance %	Cumulative %	Characteristic root	Variance %	Cumulative %	Characteristic root	Variance %	Cumulative %
1	15.524	37.863	37.863	15.524	37.863	37.863	12.046	29.381	29.381
2	5.888	14.361	52.224	5.888	14.361	52.224	5.712	13.933	43.314
3	3.810	9.292	61.516	3.810	9.292	61.516	3.264	7.961	51.275
4	1.905	4.646	66.163	1.905	4.646	66.163	2.796	6.820	58.095
5	1.630	3.977	70.139	1.630	3.977	70.139	2.536	6.185	64.280
6	1.512	3.689	73.828	1.512	3.689	73.828	2.352	5.736	70.016
7	1.465	3.572	77.400	1.465	3.572	77.400	1.990	4.852	74.869
8	1.136	2.770	80.170	1.136	2.770	80.170	1.756	4.282	79.151
9	1.067	2.602	82.772	1.067	2.602	82.772	1.485	3.621	82.772

Based on the analysis, it was found that nine common factors with a characteristic root greater than 1 were extracted through principal component analysis. Although the individual contribution of these nine common factors changed slightly after factor rotation, the cumulative value of the contribution was 82.77%, with no change before and after factor rotation. Combined with the factor loading matrix, these nine common factors were further analyzed in relation to which specific indicators, so as to further classify and downscale the indicators. As a result of the impact load factor, the load factor was greater than 0.5. Finally, 14 indicators were removed, leaving 27 indicators, which are shown in Table 3.

**Table 3:** Urban water supply system index classification.

Systems	Indicators	Unit
Water source *B*_1_	Reservoir capacity at the year-end *C*_1_^7^	100 million m^3^
	Quantity of permanent residents at the year-end *C*_2_^26^	Ten thousand people
	Urbanization rate *C*_3_^2^	%
	Water resources per capita *C*_4_^2^	m^3^/ people
	Water consumption per 10,000 RMB of industrial added value *C*_5_^6^	m^3^/ Ten thousand RMB
	Water consumption per 10,000 RMB GDP *C*_6_^7^	m^3^/ Ten thousand RMB
Water plants *B*_2_	Domestic water consumption of urban residents *C*_7_^6^	100 million m^3^
	Comprehensive production capacity of water supply *C*_8_^9^	10,000 m^3^/day
	Personnel employed in urban units in the management of water conservancy, environment and public facilities *C*_9_^9^	Ten thousand people
	Urban sewage treatment rate *C*_10_^7^	%
	Total water supply *C*_11_^14^	100 million m^3^
	Investment in waste water treatment project has been completed *C*_12_^16^	Ten thousand RMB
Water supply and distribution network		
*B*_3_	Length of water supply pipe *C*_13_^16^	Kilometre
	Density of water supply pipeline in built-up area *C*_14_^17^	km/km^2^
	Investment in fixed assets of water conservancy, environment and public facilities management industry *C*_15_^16^	100 million RMB
Users *B*_4_	The quantity of people affected by floods and droughts *C*_16_^16^	Ten thousand people
	Percentage of urban basic medical insurance coverage at year-end *C*_17_^7^	Ten thousand people
	Quantity of people enrolled in unemployment insurance *C*_18_^26^	Ten thousand people
	Quantity of community health service centers *C*_19_^28^	Individual
	State funds for education *C*_20_^26^	Ten thousand RMB
	GDP per capita *C*_21_^28^	RMB/ person
	Per capita disposable income of urban residents *C*_22_^7^	RMB/ person
	More old population dependency ratio *C*_23_^29^	%
	Urban registered unemployment rate *C*_24_^7^	%
	Natural population growth rate *C*_25_^7^	%
	Economize water consumption *C*_26_^29^	10,000 m^3^
	Water consumption exceeding the planned quota *C*_27_^31^	10,000 m^3^

By referring to the relevant toughness theory literature and combining the above factor analysis results, the subject group jointly discussed the above nine common factors as nine factors affecting the toughness of the water supply and distribution system and categorized and named them. Combined with the above toughness capability analysis framework, the following 5 index system dimensions were finally formed. Organizational—factor 1, Economic—factor 2, Natural Environment—factors 3 and 7, Physical—factors 4, 8 and 9, and Social—factors 5 and 6. For the results of the factor classification, see Table 4.

**Table 4:** Factor classification results.

Factor no	Indicators	Amount
1	*C*_2_, *C*_7_, *C*_8_, *C*_9_, *C*_13_, *C*_17_, *C*_18_, *C*_19_, *C*_20_	9
2	*C*_3_, *C*_14_, *C*_21_, *C*_22_	4
3	*C*_4_, *C*_5_, *C*_10_	3
4	*C*_15_, *C*_23_	2
5	*C*_26_, *C*_27_	2
6	*C*_24_, *C*_25_	2
7	*C*_1_, *C*_16_	2
8	*C*_6_, *C*_11_	2
9	*C*_12_	1

### Construction of toughness ability index system of urban water supply system

Based on the above composition of the urban water supply system and combined with the results of factor analysis, a system of resilience indicators for the urban water supply system is constructed, as showed in Table 5.

**Table 5:** Index system for resilience capacity assessments.

Dimension	Water source	Water plants	Water supply and distribution network	Users
Organization	*C*_2_	*C*_7_, *C*_8_, *C*_9_	*C*_13_	*C*_17_, *C*_18_, *C*_19_, *C*_20_
Economy	*C*_3_	–	*C*_14_	*C*_21_, *C*_22_
Natural environment	*C*_1_, *C*_4_, *C*_5_	*C*_10_	–	*C*_16_
Physics	*C*_6_	*C*_11_, *C*_12_	*C*_15_	*C*_23_
Social	–	–	–	*C*_24_, *C*_25_, *C*_26_, *C*_27_

## Methods for assessing resilience of the urban water supply systems

### Index weighting: entropy weight method

#### Calculation steps

1. Standardization of index data.
  This is because the units of measurement of the various indicators are not uniform and vary greatly in number^26^. In order to eliminate the influence of the different dimensions of the various indicators on programme decisions, it is necessary to standardize the indicators. Indicators are divided into two categories based on their nature. One is the larger the better, also known as positive indicators, and the other is the smaller the better indicator, also known as negative indicator. In the process of standardization, the appropriate form of standardisation should be adopted according to the nature of the indicator. Suppose I have *m* samples, *n* evaluation indicators, and *T* years of data.
  For the positive indicators:
  2 $$\overline{x}_{ijt} = \frac{{x_{ijt} - \mathop {\min }\limits_{1 \le i \le m} x_{ijt} }}{{\mathop {\max }\limits_{1 \le i \le m} x_{ijt} - \mathop {\min }\limits_{1 \le i \le m} x_{ijt} }}$$
  For the negative indicators:
  3 $$\overline{x}_{ijt} = \frac{{\mathop {\max }\limits_{1 \le i \le m} x_{ijt} - x_{ijt} }}{{\mathop {\max }\limits_{1 \le i \le m} x_{ijt} - \mathop {\min }\limits_{1 \le i \le m} x_{ijt} }}$$
  where: *x*_*ijt*_ is the original index value of index *j* in city *i* in the *t* year, and $\overline{x}_{ijt}$ is the standardized value of index *j* in city *i* in the *t* year. *i* = 1, 2, …, *m*; *j* = 1, 2, …, *n*; *t* = 1, 2, …, *T.*
2. Calculate the characteristic proportion of the assessed value of city *i* of index *j* in year *t*:
  4 $$P_{ijt} = \frac{{\overline{x}_{ijt} }}{{\sum\nolimits_{i = 1}^{m} {\overline{x}_{ijt} } }},\quad i = {1},{ 2}, \, \ldots ,{\text{ m}};j = {1},{ 2}, \, \ldots ,{\text{ n}}$$
3. Calculate the information entropy (*E*_*jt*_) and differential coefficient (*d*_*jt*_) of the *j* index in year *t*:
4. $$E_{jt} = - \frac{1}{\ln (n)}\sum\limits_{i = 1}^{m} {P_{ijt} \ln (P_{ijt} } )$$, *i* = 1, 2, …, *m*; j = 1, 2, …, *n* (5)
  6 $$d_{ijt} = 1 - E_{jt} ,\;j = 1, \, 2, \, \ldots ,n$$
  Calculate the weight of each evaluation index in year *t*:
  7 $$W_{jt} = \frac{{d_{jt} }}{{\sum\nolimits_{j = 1}^{n} {d_{jt} } }}$$
5. In order to obtain the uniform weight of each index, the average value of the sample index data in year *t* was taken and the uniform weight of the indicator was calculated using the arithmetic mean method:
  8 $$\overline{W}_{jt} = \sum\limits_{t = 1}^{T} {W_{jt} } /T$$

#### Calculation of unified weight

When determining the weights of indicators for the comprehensive evaluation of the resilience of urban water supply systems for multiple years and regions, both vertically and horizontally, the weights of the calculated indicators differed due to the different data from year to year. Consequently, the combined assessment values lack comparability when compared longitudinally, affecting the final evaluation results^27^.

Therefore, in this paper, the raw data of the resilience indicators for the supply and distribution system from 31 provinces and cities in China from 2016 to 2019, which contributed 10%, 20%, 30% and 40% to the “unified weights”, respectively, were used in the calculation of the unified weights to reflect the advantage of annual changes in the data. The results of unified weight calculation by MATLAB software (https://ww2.mathworks.cn/products/matlab.html, version 7.0) are shown in Table 6.

**Table 6:** Weight of indexes for resilience capacity assessment.

Systems	Indicators	2019 (40%)	2018 (30%)	2017 (20%)	2016 (10%)	Unified weight
*B*_1_ 0.3222	*C*_1_	0.072	0.072	0.075	0.075	0.0729
	*C*_2_	0.013	0.013	0.014	0.014	0.0133
	*C*_3_	0.011	0.011	0.012	0.012	0.0113
	*C*_4_	0.217	0.205	0.214	0.230	0.2141
	*C*_5_	0.006	0.006	0.006	0.005	0.0059
	*C*_6_	0.005	0.004	0.005	0.005	0.0047
*B*_2_ 0.1697	*C*_7_	0.005	0.005	0.005	0.005	0.0050
	*C*_8_	0.043	0.045	0.045	0.046	0.0443
	*C*_9_	0.016	0.016	0.018	0.018	0.0166
	*C*_10_	0.006	0.008	0.004	0.005	0.0061
	*C*_11_	0.040	0.040	0.042	0.042	0.0406
	*C*_12_	0.061	0.055	0.057	0.048	0.0571
*B*_3_ 0.1113	*C*_13_	0.046	0.045	0.049	0.050	0.0467
	*C*_14_	0.039	0.031	0.030	0.031	0.0340
	*C*_15_	0.035	0.029	0.027	0.025	0.0306
*B*_4_ 0.3974	*C*_16_	0.005	0.006	0.017	0.011	0.0083
	*C*_17_	0.036	0.046	0.048	0.041	0.0419
	*C*_18_	0.044	0.043	0.045	0.045	0.0440
	*C*_19_	0.028	0.028	0.030	0.031	0.0287
	*C*_20_	0.029	0.028	0.028	0.029	0.0285
	*C*_21_	0.034	0.037	0.039	0.045	0.0370
	*C*_22_	0.058	0.062	0.064	0.065	0.0611
	*C*_23_	0.020	0.017	0.017	0.013	0.0178
	*C*_24_	0.022	0.030	0.017	0.017	0.0229
	*C*_25_	0.020	0.020	0.017	0.014	0.0188
	*C*_26_	0.076	0.090	0.066	0.069	0.0775
	*C*_27_	0.015	0.006	0.011	0.009	0.0109

### A cloud model for assessing resilience of urban water supply systems

#### Cloud model construction steps

When determining the membership degree, the traditional fuzzy membership degree is a fixed value. However, when using a cloud model to calculate the membership of indicators in the cloud, the membership of the indicators in the evaluation set is inaccurate and unique, thus reducing the subjectivity and difficulty^28^.

Establishment steps of a multi-level comprehensive evaluation model based on a cloud model^29,30^:

1. Create a factor field *U* and comment field *V* for the evaluation objects.
2. The calculated index weights *W* were adopted.
3. A single factor evaluation was conducted between *U* and *V* A fuzzy relational matrix *R* was established. Let factor *i*(*i* = 1, 2, …, *n*) corresponding grade *j*(*j* = 1, 2, …, *m*)have an upper boundary value is *x*_*ij*_ and the lower boundary value is *x’*_*ij*_, then the qualitative concept of level j corresponding to factor I can be represented by a normal cloud model, where:
  9 $$Ex_{ij} = (x_{ij} + x^{,}_{ij} )/2$$
  Since the boundary values are the transition value of two adjacent levels and the degree of membership of the two levels are equal, there are:
  10 $$\exp \left\{ { - \frac{{(x_{ij} - x^{,}_{ij} )^{2} }}{{8(En_{ij} )^{2} }}} \right\} = 0.5$$
  Namely
  11 $$En_{ij} = (x_{ij} - x^{,}_{ij} )/2.355$$
4. The cloud model membership matrix *C’* = (*c*_*ij*_)_*n*×*m*_ for each index corresponding to each metric at the system layer is calculated based on the metric values of the evaluated object, where *C*_*ij*_ is the average value under different membership degrees (normal cloud generator under *X* condition is run *N* times) :
  12 $$C_{ij} = \frac{1}{N}\sum\limits_{k = 1}^{n} {C_{ij}^{k} }$$
5. The fuzzy subset *B’* on the evaluation set *V* of the system layer is obtained through the fuzzy transformation between the weight set *W’* of the indicator layer and the membership matrix *C’* :
  13 $$B^{^{\prime}} = W^{\prime}*C^{\prime} = (b_{1} b_{2} \cdots b_{m} )$$
  In the formula:
  14 $$b_{j} = \sum\limits_{i = 1}^{n} {w_{i} c_{ij} j = 1 \cdots m}$$
  *b*_*j*_ represents the membership degree of the object to be evaluated to the comments in Article *j*. According to the principle of maximum membership degree, the *i* evaluation grade corresponding to the maximum membership degree of article *j* comments was selected as the result of system-level evaluation*.*
6. Similarly, the fuzzy subset *B* of the target layer is obtained by a high-level fuzzy transformation between the set of weights *W* of the system layer and the fuzzy subset *B’* of the system layer. Finally, the comprehensive evaluation level of the target layer is obtained according to the principle of maximum membership.

## Case analysis

### Study areas and data source

Qingdao is one of the regions in northern China with serious water shortage. Water resources are inherently insufficient and unevenly distributed in time and space, and the conflict between supply and demand is becoming increasingly prominent. Precipitation is characterized by large intra-annual and inter-annual variation and is prone to droughts and floods. Located on the Jiaodong Peninsula, it is susceptible to high winds, heavy rainfall and tidal weather. With the rapid development of economy and society and the increasing level of urbanization, water supply security has become a “bottleneck” factor limiting the sustainable development of Qingdao’s economy and society.

During the 12th Five-year Plan period, Qingdao invested 13.82 billion RMB to build a large water supply system with “three major water sources” raw water supply, the “four verticals and three horizontals” pipe networks for transmission and distribution in the main city, and “one ring and three lines” for unified deployment in Qingdao.

This paper takes Qingdao as an example. Data are obtained from 2011–2020 “Qingdao Water Resources Bulletin”, “Qingdao Statistical Yearbook”, “Qingdao Statistical Bulletin”, “Shandong Province Statistical Yearbook” and so on.

**Figure 6:**
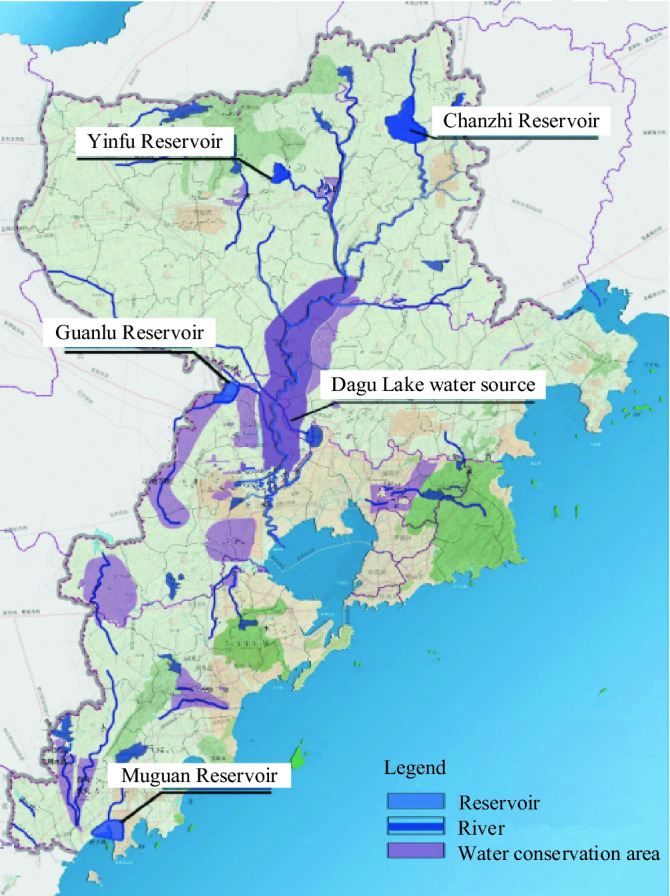
Distribution of water resources in Qingdao (the figure shows the distribution of reservoirs, rivers and water conservation areas in Qingdao).

At present, the distribution of water resources in Qingdao is shown in Fig. 6 (ed2k://|file|SW_DVD5_Visio_Pro_2016_64Bit_ChnSimp_MLF_X20-42,759.ISO|714,913,792|FC930AB97B366B3595FC2F28ABAC2A6F|/ , version Visio_Pro_2016_64Bit). The current status of the main water supply projects in Qingdao is shown in Table 7.

**Table 7:** Present situation of the main water source and water supply project in Qingdao (data from Qingdao Water Resources Construction and Allocation “Thirteenth Five-Year Plan”).

No	Water source	Water plants	Water supply area	Water supply capacity*(10,000 m^3^/d)
1	Chanzhi reservoir	Zhangezhuang waterworks	Laixi city	10
2	Chanzhi reservoir	Baishahe waterworks	Five districts in Qingdao	8
3	Yinfu reservoir	Xingping waterworks	Pingdu city	2
4	Yinfu reservoir	Baishahe waterworks	Five districts in Qingdao	4
5	Huangshan reservoir	Xingping waterworks	Pingdu city	0.3
6	Zhangling water source	Yunshan waterworks	Pingdu city	2.2
7	Wangquan reservoir	Shibei waterworks	Jimo city	2
8	Songhuaquan reservoir	Shibei waterworks	Jimo city	0.2
9	Nuocheng reservoir	Tongji waterworks	Jimo city	12
10	Shipeng reservoir	Shinan waterworks	Jimo city	2
11	Moshuihe water source	Wuqi waterworks	Jimo city	1
12	Water source	Baishahe waterworks	Five districts in Qingdao	10
13	Qingnian reservoir	Zhuanglitou waterworks	Jiaozhou city	0.5
14	Shanzhou reservoir	Zhuanglitou waterworks	Jiaozhou city	1
15	Shuyuan reservoir	Jiangjiazhuang waterworks	Five districts in Qingdao	1.5
16	Laoshan reservoir	Laoshan waterworks	Five districts in Qingdao	7.5
17	Baishahe water source	Xiazhuang waterworks	Five districts in Qingdao	3.5
	Shuanglong waterworks			
18	Xiaozhushan reservoir	Xiaozhushan waterworks	Huangdao district	2
19	Jilihe reservoir	Gaojiatai waterworks	Huangdao district	5.5
20	Tieshan reservoir	No.3 waterworks	Huangdao district	2
21	Douyazi reservoir	No.5 waterworks	Huangdao district	6
22	Fenghe water source	No.2 and No.4 waterworks	Huangdao district	5.8
23	Jihongtan reservoir	Xianjiazhai waterworks	Five districts in Qingdao	23.5
24	Jihongtan reservoir	Kaifaqu waterworks	Jiaozhou city	1.5
25	Jihongtan reservoir	Guanjialou waterworks	Huangdao district	10
26	Jihongtan reservoir	Hongshiya waterworks	Huangdao district	16
27	Jihongtan reservoir	Western water supply office	Five districts in Qingdao	3
28	Chanzhi reservoir	Huashan waterworks	Jimo city	10
		Huangjiashan waterworks	Chengyang district	

In order to ensure the objectivity and comparability of the evaluation criteria of the indicators. In this paper, based on the sample data of 31 Chinese provinces and cities and the indicator values of the evaluation objects, the evaluation criteria of the resilience evaluation indicators of the evaluation objects are developed, as shown in Table 8.

**Table 8:** Toughness evaluation index standard.

Index	Low level	Slightly lower level	Medium level	Slightly higher level	High level
*C*_1_	0, 1	1, 2	2, 3	3, 4	4, 5
*C*_2_	1050, 1000	1000, 950	950, 900	900, 850	850, 800
*C*_3_	25, 40	40, 55	55, 70	70, 85	85,100
*C*_4_	0, 500	500, 1000	1000, 3000	3000, 5000	5000, 7000
*C*_5_	130, 90	90, 50	50, 10	10, 5	5, 0
*C*_6_	150, 100	100, 60	60, 30	30, 15	15, 0
*C*_7_	30,000, 20,000	20,000, 15,000	15,000, 10,000	10,000, 5000	5000, 0
*C*_8_	0, 100	100, 200	200, 300	300, 400	400, 500
*C*_9_	0, 1	1, 2	2, 3	3, 4	4, 5
*C*_10_	55, 65	65, 75	75, 85	85, 95	95, 100
*C*_11_	0, 30,000	30,000, 60,000	60,000, 90,000	90,000, 120,000	120,000, 150,000
*C*_12_	0, 5000	5000, 10,000	10,000, 50,000	50,000, 100,000	100,000, 200,000
*C*_13_	0, 2000	2000, 4000	4000, 6000	6000, 8000	8000, 10,000
*C*_14_	0, 5	5, 10	10, 20	20, 30	30, 40
*C*_15_	0, 150	150, 300	300, 450	450, 600	600, 750
*C*_16_	30,000, 24,000	24,000, 18,000	18,000, 12,000	12,000, 6000	6000, 0
*C*_17_	0, 20	20, 40	40, 60	60, 80	80, 100
*C*_18_	0, 60	60, 120	120, 180	180, 240	240, 300
*C*_19_	0, 40	40, 60	60, 80	80, 100	100, 120
*C*_20_	0, 1,000,000	1,000,000, 2,000,000	2,000,000, 3,000,000	3,000,000, 4,000,000	4,000,000, 5,000,000
*C*_21_	0, 30,000	30,000, 60,000	60,000, 90,000	90,000, 120,000	120,000, 150,000
*C*_22_	0, 14,000	14,000, 28,000	28,000, 42,000	42,000, 56,000	56,000, 70,000
*C*_23_	25, 20	20, 15	15, 10	10, 5	5, 0
*C*_24_	5, 4	4, 3	3, 2	2, 1	1, 0
*C*_25_	10, 8	8, 6	6, 4	4, 2	2, 0
*C*_26_	0, 5000	5000, 10,000	10,000, 20,000	20,000, 30,000	30,000, 40,000
*C*_27_	3000, 1500	1500, 900	900, 700	700, 500	500, 0

According to the established toughness index system and evaluation index standard of regional water supply system, the ranking criteria corresponding to each index can be represented by the normal cloud model corresponding to Eqs. (9) and (11), as shown in Table 9.

**Table 9:** Toughness evaluation index normal cloud standard.

Index	Low level	Slightly lower level	Medium level	Slightly higher level	High level
*C*_1_	0.5, 0.42, 0.1	1.5, 0.42, 0.1	2.5, 0.42, 0.1	3.5, 0.42, 0.1	4.5, 0.42, 0.1
*C*_2_	1025 , 21.23, 5	975 , 21.23, 5	925, 21.23, 5	875, 21.23, 5	825, 21.23, 5
*C*_3_	32.5, 6.37, 0.5	47.5, 6.37, 0.5	62.5, 6.37, 0.5	77.5, 6.37, 0.5	92.5, 6.37, 0.5
*C*_4_	250, 212.31, 10	750, 212.31, 10	2000, 849.26, 50	4000, 849.26, 50	6000, 849.26, 50
*C*_5_	110, 16.99, 2	70, 16.99, 2	30, 16.99, 2	7.5, 2.12, 0.2	2.5, 2.12, 0.2
*C*_6_	125, 21.23, 2	80, 16.99, 2	45, 12.74, 2	22.5, 6.37, 1.5	7.5, 6.37, 1.5
*C*_7_	25,000, 4246.28, 100	17,500, 2123.14, 100	12,500, 2123.14, 100	7500, 2123.14, 100	2500, 2123.14, 100
*C*_8_	50, 42.46, 5	150, 42.46, 5	250, 42.46, 5	350, 42.46, 5	450, 42.46, 5
*C*_9_	0.5, 0.42, 1	1.5, 0.42, 1	2.5, 0.42, 1	3.5, 0.42, 1	4.5, 0.42, 1
*C*_10_	60, 4.25, 1	70, 4.25, 1	80, 4.25, 1	90, 4.25, 1	97.5, 2.12, 1
*C*_11_	15,000, 12,738.85, 2000	45,000, 12,738.85, 2000	75,000, 12,738.85, 2000	105,000, 12,738.85, 2000	135,000, 12,738.85, 2000
*C*_12_	2500, 2123.14, 50	7500, 2123.14, 50	30,000, 16,985.14, 50	75,000, 21,231.42, 50	150,000, 42,462.85, 50
*C*_13_	1000, 846.25, 100	3000, 846.25, 100	5000, 846.25, 100	7000, 846.25, 100	9000, 846.25, 100
*C*_14_	2.5, 2.12, 0.5	7.5, 2.12, 0.5	15, 4.25, 0.5	25, 4.25, 0.5	35, 4.25, 0.5
*C*_15_	75, 63.69, 10	225, 63.69, 10	375, 63.69, 10	525, 63.69, 10	675, 63.69, 10
*C*_16_	27,000, 2547.77, 250	21,000, 2547.77, 250	15,000, 2547.77, 250	9000, 2547.77, 250	3000, 2547.77, 250
*C*_17_	10, 8.49, 1.5	30, 8.49, 1.5	50, 8.49, 1.5	70, 8.49, 1.5	90, 8.49, 1.5
*C*_18_	30, 25.48, 5	90, 25.48, 5	150, 25.48, 5	210, 25.48, 5	270, 25.48, 5
*C*_19_	20, 16.99, 2.5	50, 16.99, 2.5	70, 16.99, 2.5	90, 16.99, 2.5	110, 16.99, 2.5
*C*_20_	500,000, 424,628.5,5000	1,500,000, 424,628.5,5000	2,500,000, 424,628.5,5000	3,500,000, 424,628.5,5000	4,500,000, 424,628.5,5000
*C*_21_	15,000, 12,738.85, 2000	45,000, 12,738.85, 2000	75,000, 12,738.85, 2000	105,000, 12,738.85, 2000	135,000, 12,738.85, 2000
*C*_22_	7000, 5944.8, 800	21,000, 5944.8, 800	35,000, 5944.8, 800	49,000, 5944.8, 800	63,000, 5944.8, 800
*C*_23_	22.5, 2.12, 0.4	17.5, 2.12, 0.4	12.5, 2.12, 0.4	7.5, 2.12, 0.4	2.5, 2.12, 0.4
*C*_24_	4.5, 0.42, 0.1	3.5, 0.42, 0.1	2.5, 0.42, 0.1	1.5, 0.42, 0.1	0.5, 0.42, 0.1
*C*_25_	9, 0.85, 0.2	7, 0.85, 0.2	5, 0.85, 0.2	3, 0.85, 0.2	1, 0.85, 0.2
*C*_26_	2500, 2123.14 , 50	7500, 2123.14, 50	15,000, 4246.28, 50	25,000, 4246.28, 50	35,000, 4246.28, 50
*C*_27_	2250, 636.94, 30	1200, 254.78, 30	800, 84.93, 20	600, 84.93, 20	250, 212.31, 30

### Multi-level comprehensive evaluation based on the cloud model

According to the established toughness capability index system and index standard, a normal cloud model is used to represent the ranking standard of each index in Eqs. (9) and (11).

**Figure 7:**
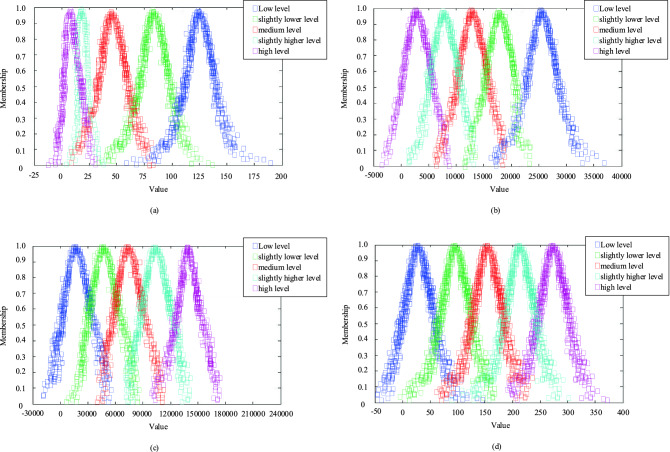
Membership functions for Normal cloud (**a**) take *C*_6_ as an example, (**b**) take *C*_7_ as an example, (**c**) take *C*_11_ as an example, (**d**) take *C*_18_ as an example).

Taking index *C*_6_, *C*_7_, *C*_11_, *C*_18_ as examples, using Eq. (1) and cloud matrix *R* (Table 9), a normal cloud membership function of evaluation index standard can be established through the positive normal cloud generator, as shown in Fig. 7.

**Figure 8:**
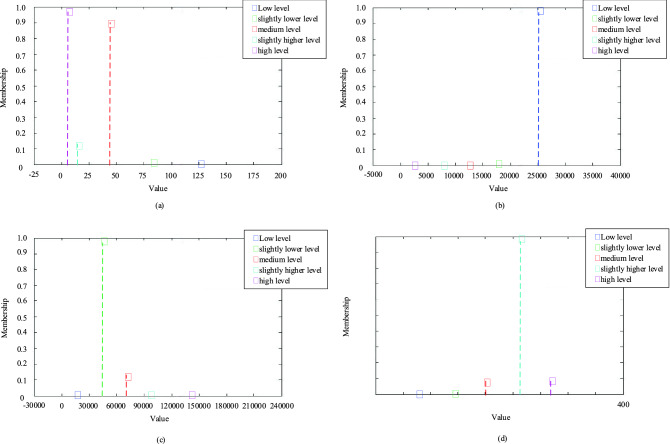
Average membership degree of index under *X*-conditional normal cloud generator.

As can be seen from Fig. 7, the simulation results obtained by selecting different metrics vary considerably. However, it is obvious that the membership function of the normal cloud, which represents a medium level is relatively stable and always in the middle. Therefore, it can be intuitively assumed that the resilience of the water supply system in Qingdao is at a medium level. Taking indices *C*_6_, *C*_7_, *C*_11_, *C*_18_ as examples, the number of cloud drops generated is assumed to be *N* = 800, and the X conditional cloud generator algorithm is used to generate the membership matrix according to the corresponding index value. Taking in the case of the Qingdao water supply system, the data of Qingdao from 2011 to 2020 are substituted into the *X*-conditional normal cloud generator composed of the above evaluation level cloud model, and the average membership of each evaluation level is calculated 800 times (Fig. 8 and Table 10).

**Table 10:** Evaluation index: average membership of normal cloud.

Index	Low level	Slightly lower level	Medium level	Slightly higher level	High level
*C*_1_	0.0057	0.4166	0.5155	0.0102	0.0000
*C*_2_	0.0008	0.1122	0.9779	0.0601	0.0004
*C*_3_	0.0000	0.0007	0.2841	0.7390	0.0090
*C*_4_	0.8686	0.0162	0.0923	0.0000	0.0000
*C*_5_	0.0000	0.0014	0.3215	0.3868	0.6118
*C*_6_	0.0000	0.0005	0.8845	0.1095	0.9833
*C*_7_	0.9931	0.0009	0.0000	0.0000	0.0000
*C*_8_	0.0000	0.4824	0.5000	0.0037	0.0000
*C*_9_	0.0000	0.0014	0.1761	0.8667	0.0331
*C*_10_	0.0000	0.0000	0.0029	0.2536	0.9114
*C*_11_	0.0500	0.9829	0.1034	0.0003	0.0000
*C*_12_	0.0000	0.0000	0.1852	0.8095	0.1123
*C*_13_	0.0000	0.0006	0.1998	0.8471	0.0173
*C*_14_	0.0071	0.4386	0.5183	0.0037	0.0000
*C*_15_	0.0000	0.0044	0.4589	0.5199	0.0057
*C*_16_	0.0411	0.9785	0.1010	0.0001	0.0000
*C*_17_	0.0000	0.0000	0.0002	0.0576	0.9920
*C*_18_	0.0000	0.0003	0.0748	0.9999	0.0812
*C*_19_	0.0171	0.4573	0.9981	0.5222	0.0803
*C*_20_	0.0000	0.0505	0.9951	0.0766	0.0000
*C*_21_	0.0000	0.0000	0.0058	0.5231	0.4509
*C*_22_	0.0000	0.0000	0.1323	0.9516	0.0370
*C*_23_	0.5652	0.4051	0.0045	0.0000	0.0000
*C*_24_	0.0132	0.6003	0.3463	0.0042	0.0000
*C*_25_	0.0017	0.1868	0.8743	0.0337	0.0002
*C*_26_	0.0064	0.7088	0.4017	0.0010	0.0000
*C*_27_	0.0517	0.1408	0.4249	0.5331	0.1249

**Figure 9:**
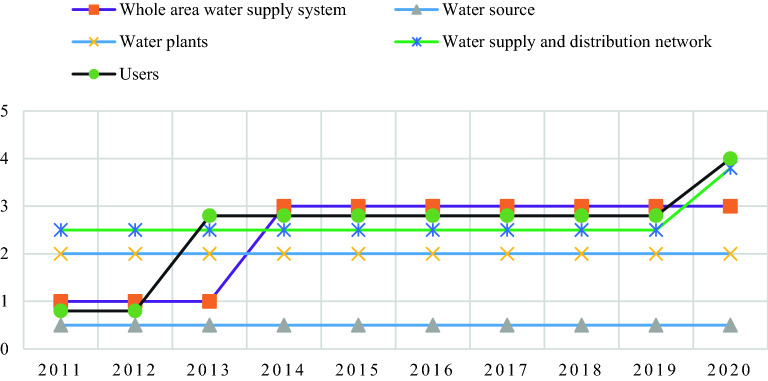
Trends in cloud assessment levels of resilience capacity.

Finally, according to steps (5) and (6) of the cloud model, comprehensive evaluation results are obtained, as shown in Table 11 and Fig. 9. To ensure the completeness of the evaluation, the entire water supply system (*B*_0_) is added as one of the toughness evaluation indexes.

**Table 11:** Comprehensive assessment results of resilience capacity.

Year	Systems	Low level	Slightly lower level	Medium level	Slightly higher level	High level	Rating
2020	*B*_0_	0.069	0.063	0.087	0.079	0.032	Medium level
	*B*_1_	0.194	0.035	0.080	0.013	0.008	Low level
	*B*_2_	0.007	0.062	0.040	0.061	0.012	Slightly lower level
	*B*_3_	0.000	0.015	0.040	0.056	0.001	Slightly higher level
	*B*_4_	0.011	0.101	0.128	0.148	0.068	Slightly higher level
2019	*B*_0_	0.077	0.049	0.087	0.073	0.024	Medium level
	*B*_1_	0.224	0.005	0.043	0.016	0.005	Low level
	*B*_2_	0.007	0.076	0.050	0.042	0.008	Slightly lower level
	*B*_3_	0.000	0.029	0.049	0.029	0.000	Medium level
	*B*_4_	0.006	0.081	0.150	0.148	0.053	Medium level
2018	*B*_0_	0.079	0.056	0.101	0.052	0.022	Medium level
	*B*_1_	0.229	0.007	0.040	0.014	0.007	Low level
	*B*_2_	0.008	0.074	0.070	0.015	0.004	Slightly lower level
	*B*_3_	0.000	0.034	0.066	0.030	0.000	Medium level
	*B*_4_	0.006	0.095	0.175	0.105	0.050	Medium level
2017	*B*_0_	0.068	0.086	0.144	0.050	0.007	Medium level
	*B*_1_	0.197	0.064	0.130	0.016	0.005	Low level
	*B*_2_	0.007	0.081	0.024	0.063	0.014	Slightly lower level
	*B*_3_	0.001	0.028	0.051	0.031	0.000	Medium level
	*B*_4_	0.006	0.123	0.234	0.077	0.007	Medium level
2016	*B*_0_	0.071	0.065	0.125	0.055	0.009	Medium level
	*B*_1_	0.202	0.005	0.046	0.085	0.014	Low level
	*B*_2_	0.011	0.085	0.069	0.006	0.002	Slightly lower level
	*B*_3_	0.000	0.025	0.075	0.011	0.000	Medium level
	*B*_4_	0.007	0.118	0.230	0.063	0.011	Medium level
2015	*B*_0_	0.070	0.073	0.122	0.031	0.023	Medium level
	*B*_1_	0.197	0.004	0.039	0.042	0.052	Low level
	*B*_2_	0.010	0.085	0.055	0.007	0.001	Slightly lower level
	*B*_3_	0.002	0.030	0.082	0.008	0.000	Medium level
	*B*_4_	0.009	0.138	0.231	0.037	0.014	Medium level
2014	*B*_0_	0.084	0.090	0.108	0.018	0.032	Medium level
	*B*_1_	0.222	0.013	0.044	0.027	0.067	Low level
	*B*_2_	0.014	0.095	0.047	0.003	0.005	Slightly lower level
	*B*_3_	0.006	0.025	0.076	0.012	0.000	Medium level
	*B*_4_	0.020	0.171	0.196	0.018	0.023	Medium level
2013	*B*_0_	0.084	0.078	0.082	0.015	0.035	Low level
	*B*_1_	0.177	0.003	0.020	0.028	0.077	Low level
	*B*_2_	0.013	0.096	0.050	0.006	0.000	Slightly lower level
	*B*_3_	0.002	0.032	0.079	0.010	0.000	Medium level
	*B*_4_	0.058	0.145	0.148	0.008	0.023	Medium level
2012	*B*_0_	0.099	0.076	0.071	0.035	0.013	Low level
	*B*_1_	0.179	0.003	0.040	0.081	0.021	Low level
	*B*_2_	0.015	0.121	0.046	0.003	0.000	Slightly lower level
	*B*_3_	0.002	0.033	0.077	0.009	0.000	Medium level
	*B*_4_	0.096	0.130	0.107	0.018	0.016	Slightly lower level
2011	*B*_0_	0.108	0.082	0.081	0.016	0.018	Low level
	*B*_1_	0.213	0.028	0.099	0.034	0.011	Low level
	*B*_2_	0.016	0.073	0.018	0.002	0.053	Slightly lower level
	*B*_3_	0.000	0.018	0.072	0.008	0.000	Medium level
	*B*_4_	0.090	0.149	0.095	0.010	0.012	Slightly lower level

The comprehensive evaluation results show that the resilience level of Qingdao's water supply network is at a medium level from 2014 to 2020, and at a low level in 2011, 2012 and 2013. The overall development of toughness level is favorable. Of all the water supply subsystems, the resilience of the water source system has been at a low level and has not been improved in the last decade. In 2011 and 2012, the resilience of the customer system was at a sluggish level. It was at a medium level from 2013 to 2019 and at a high level in 2020, showing a gradual upward trend. Over the past decade, the toughness of the water plant system has been at a low level. The resilience of water supply and distribution network system has been stable at a medium level from 2011 to 2019 and increased to a higher level in 2020. Since 2013, the toughness of all subsystems in order is: the toughness of user system and water supply network system is the same, ranking the highest, followed by water plant system and water source system.

In recent years, Qingdao has experienced a rapid level of economic development and a high level of social service security. These two factors mainly reflect the resilience of the user system. As a result, the resilience of the user system shows a step-up upward trend. In September 2019, the Qingdao municipal government issued the “13th Five-year Plan” for water source construction and allocation, in which water source construction projects mainly include reservoirs, pond dams, river gates and other water supply projects such as rainwater collection projects, recycled water utilization and seawater use. Water allocation projects refer to the pipeline (underground channels, open channels, pipelines) and supporting pumping stations and other water transmission infrastructure from the water intake to the waterworks. The short construction cycle of the water supply and distribution pipelines is evident, in line with the change of toughness evaluation grade of water supply and distribution pipeline network. The toughness rating has increased from the medium level in 2019 to the high level in 2020. Water sources and plants have a long construction period and will not be able to deliver real benefits until after they are operational. Therefore, the resilience levels for water sources and plants have a long growth period and can be verified in conjunction with the evaluation results in 2019 and 2020. Although the resilience of users and water supply and distribution network system has been improved in 2019, their contribution to the overall toughness level of the water supply system was limited, and the overall toughness of the water supply system remained at a medium level in 2019. The study found that the low level of toughness of the water source system is the main reason for this phenomenon, and that water shortage has been the shortcoming in the development of the overall resilience of Qingdao's water supply system.

### Calculation of resilience capacity of index comprehensive evaluation index method based on entropy weight

As most previous studies have used a comprehensive evaluation index method based on entropy weight to calculate resilience, this method is applied to this case to observe whether there is a large gap in the calculation results.

The weights of the regional water supply and distribution system are calculated using a uniform weight value *W*_*j*_ with entropy weights and a toughness evaluation index system of the regional urban water supply system. The evaluation indexes go through the process of data collection, input and data standardization.

Here, the raw index data is standardized by the extremum method (*min–max* normalization method) to eliminate the dimensional effects.

**Figure 10:**
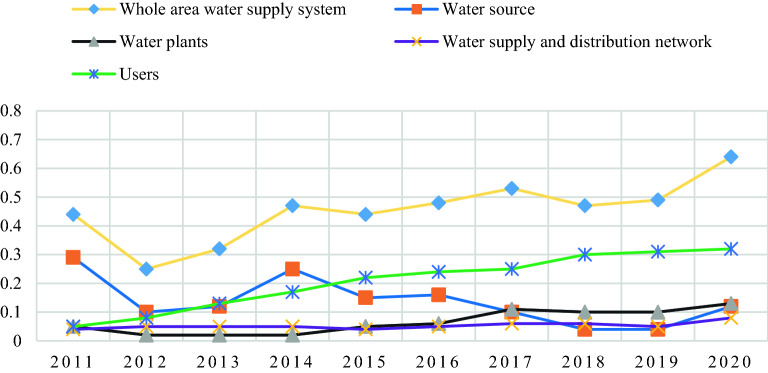
Comprehensive evaluation index trend of toughness capability by entropy weight method.

The normalized index *r*_*ij*_ of evaluation indexes *j* in the *i* year was calculated, and then the comprehensive evaluation index *V* = *W*_*j*_ * r_*ij*_ was synthesized. In the case of 2020, the calculation process is similar for the other years. The final toughness capacity calculation results are shown in Table 12 and Fig. 10.

**Table 12:** Toughness capability evaluation indexes are comprehensive evaluation index.

Systems	Index	Standardized values	Index weight	Composite
*B*_1_ 0.3300	*C*_1_	0.4013	0.0706	0.0283
	*C*_2_	0.0000	0.0140	0.0000
	*C*_3_	1.0000	0.0121	0.0121
	*C*_4_	0.2621	0.2226	0.0583
	*C*_5_	0.9464	0.0057	0.0054
	*C*_6_	1.0000	0.0049	0.0049
*B*_2_ 0.1689	*C*_7_	0.0000	0.0051	0.0000
	*C*_8_	1.0000	0.0448	0.0448
	*C*_9_	0.9231	0.0175	0.0162
	*C*_10_	1.0000	0.0053	0.0053
	*C*_11_	0.9529	0.0414	0.0395
	*C*_12_	0.4000	0.0548	0.0219
*B*_3_ 0.1098	*C*_13_	1.0000	0.0487	0.0487
	*C*_14_	0.0000	0.0331	0.0000
	*C*_15_	1.0000	0.0280	0.0280
*B*_4_ 0.3912	*C*_16_	0.0000	0.0086	0.0000
	*C*_17_	1.0000	0.0414	0.0414
	*C*_18_	1.0000	0.0439	0.0439
	*C*_19_	1.0000	0.0304	0.0304
	*C*_20_	1.0000	0.0279	0.0279
	*C*_21_	1.0000	0.0393	0.0393
	*C*_22_	1.0000	0.0602	0.0602
	*C*_23_	0.0000	0.0154	0.0000
	*C*_24_	0.1944	0.0236	0.0046
	*C*_25_	0.2765	0.0174	0.0048
	*C*_26_	0.6667	0.0734	0.0489
	*C*_27_	0.9080	0.0098	0.0089

As can be seen from Fig. 10, the resilience of Qingdao's water supply and distribution system shows a wave-like upward trend during the period 2011–2020, although it shows a decline in some years. It can also be seen in the figure above, the trend in the resilience index of the water supply subsystems is that the overall index of the water supply subsystem shows a downward trend throughout the year and a significant initial decline in the last year, the overall index of the customer subsystem shows an increase and growth, the water plant subsystem has been on a downward and upward trend, and for the water distribution network system there is a slight upward trend with an insignificant growth.

### Comparison of the two evaluation results

Comparing the results of the multi-level integrated evaluation based on the cloud model with the entropy-based index integrated evaluation index method, we also get:

1. The entropy-weighted index-based comprehensive evaluation index method calculates a fixed comprehensive index value that is positively correlated with the index weights. Although it reflects the overall dynamic trends of the water supply system and its sub-systems, it is difficult to describe the details of the degree of each evaluation unit belonging to a particular level. For example, in this example, the index weight for water resources per capita is large. If calculated using the entropy-weighted index-based integrated evaluation index method, it has a significant positive impact on the resilience of the system; if calculated using the cloud model's multi-level integrated evaluation model, its level of resilience is at a lower level, which has a significant but negative impact on the resilience of the system.
2. The results of multi-level comprehensive evaluation based on the cloud model are more flexible. The evaluation results examine the extent to which an evaluation unit falls within a certain level, and the boundaries of the level also change within a certain acceptable range.
3. The individual subsystem in the water supply system determines the toughness of a water supply system, but the overall toughness of the water supply system is not simply superimposed by each subsystem, but rather the interaction and interaction between individual subsystems. Therefore, the toughness ability is more in line with the fuzzy connotation of cloud model theory.

## Discussion

The water supply system is a complex mega-system, with each subsystem working independently and interrelated, and its overall toughness is the result of the joint action of multiple factors and systems working together. The toughness of the water supply system is closely related to the weight of the evaluation object and its evaluation grade. From the above evaluation results, it can be seen that the user subsystem has the highest weight and the highest evaluation rating. The water source subsystem has the second highest weighting but the lowest evaluation rating. The water plant subsystem has the third highest weight and a low evaluation level. The water supply and distribution network subsystem has the lowest weight, but the evaluation level is at a medium level. Therefore, the comprehensive ranking of the influence degree of each subsystem on the disaster toughness of the water supply system is water source subsystem, user subsystem, water plant subsystem and water supply and distribution pipe network subsystem. According to the weights of the evaluation indicators, the top 12 main indicators are per capita water resources 0.2226, water conservation 0.0734, reservoir storage at the end of the year 0.0706, per capita disposable income of urban residents 0.0602, completed investment in wastewater treatment project 0.0548, length of water supply pipeline 0.0487, comprehensive production capacity of water supply 0.0448, number of people participating in unemployment insurance 0.0439, total water supply 0.0414,proportion of basic urban medical insurance at the end of the year 0.0414, GDP per capita 0.0393, density of water supply pipes in built-up areas 0.0331. A comparative analysis shows that the main factors affecting the resilience of water supply systems can be summarized in four areas, water resources conditions, economic development level, organization and social security capacity, and key water supply infrastructure.

### Promotion strategy

Resilience strategy is not simply a strategy for resisting disasters, but a strategy that can consolidate and improve the resilience of the system to cope with various unexpected disasters, adapt to them and learn from them. In the following, the four main factors affecting the resilience of the water supply systems (water resource conditions, level of economic development, organisational and social security capacity, and critical water supply infrastructure) are discussed in relation to structural and non-structural measures to manage system resilience strategies:

1. Abandon the development model of “pollution first, treatment later, treatment while polluting” and adhere to the three red lines of development, the bottom line of ecological function protection, the upper limit of natural resource use and the bottom line of ecological security.
2. As urbanization accelerates, the demand for water in suburbs and towns is gradually increasing, which poses a serious challenge to the integration of urban and rural water supplies. Therefore, while ensuring economic growth in urban areas, investment in suburbs and towns should be increased and new urbanization should be vigorously promoted in order to narrow the internal differences in regional economies.
3. Implement dual control of water supply and water use, improve water use efficiency, and make major water use efficiency indicators such as water consumption per 10,000 yuan of GDP and water consumption per 10,000 yuan of industrial added value reach leading domestic and international advanced levels.
4. Insisting on the deployment of regional water sources, strengthening the construction of water supply pipeline networks, enhancing the connection between the municipal water supply backbone network and the regional water supply pipeline network, enhancing the resilience of the water supply system and ensuring the safety of water supply throughout the region.

## Conclusion

The resilience assessment of urban water supply systems under the influence of flood and drought disaster scenarios is studied. Based on Pearson correlation analysis and factor analysis, this paper constructs a resilience evaluation index system for urban water supply systems. The entropy weight method is used to determine the uniform weight value of each index, and the comprehensive evaluation results of the resilience of urban water supply system based on the cloud model multi-level comprehensive evaluation method and the entropy weight comprehensive index method are given. And on the basis of the evaluation, the influencing factors of the resilience of urban water supply system are analyzed. Finally, in order to ensure the sustainable improvement of the resilience of urban water supply system, the resilience improvement strategies are discussed from different dimensions, which provide decision support for further improving the disaster prevention and mitigation planning of water supply system and enhancing the resilience of the water supply system.

## Supplementary Information

Supplementary Information.
