# Supplementary material for: Evaluation and promotion strategy of resilience of urban water supply system under flood and drought disasters
Source: Sci Rep. 2022 May 6;12:7404. doi: 10.1038/s41598-022-11436-w (PMC9076867; doi:10.1038/s41598-022-11436-w)
Supplement: Supplementary file 1 — Supplementary Information. [file 41598_2022_11436_MOESM1_ESM.doc]

**Appendix 1. Source and calculation formula of ductile capacity of water supply system**

| **Index Name** | **Source of indicators** | **Data Source** | **Calculation formula** |
| --- | --- | --- | --- |
| Reservoir capacity at the year-end *C_1_* | National Annual Report of Water Situation Statistics | National Annual Report of Water Situation Statistics | Direct access |
| The quantity of people affected by floods and droughts *C_16_* | Toughness City: Risk Management and Indicators Construction-Zhou Limin | China Water and Drought Disaster Bulletin | Direct access available |
| Quantity of permanent residents at the year-end *C_2_* | The National Bureau of Statistics Website | The National Bureau of Statistics Website | Direct access available |
| Domestic water consumption of urban residents *C_7_* | China Urban Construction Statistics Yearbook | China Urban Construction Statistics Yearbook | Water Consumption for Public Service + Residential Household Water Consumption |
| Comprehensive production capacity of water supply *C_8_* | The National Bureau of Statistics Website | The National Bureau of Statistics Website | Direct available |
| Personnel employed in urban units in the management of water conservancy, environment and public facilities *C_9_* | Study on Elastic Capacity Assessment of Urban Water Supply System under the Impact of Salt Tide Situation-Liu Jian | The National Bureau of Statistics Website | Direct access available |
| Length of water supply pipe *C_13_* | China Urban Construction Statistics Yearbook | China Urban Construction Statistics Yearbook | Avavailable directly |
| Percentage of urban basic medical insurance coverage at year-end *C_17_* | Study on China Urban Disaster Resilibility Assessment and Its Improvement Strategy-Li Ya | The National Bureau of Statistics Website | Direct access available |
| Quantity of people enrolled in unemployment insurance *C_18_* | Study on China Urban Disaster Resilibility Assessment and Its Improvement Strategy-Li Ya | The National Bureau of Statistics Website | Direct access available |
| Quantity of community health service centers *C_19_* | The National Bureau of Statistics Website | The National Bureau of Statistics Website | Direct access |
| State funds for education *C_20_* | Toughness City: Risk Management and Indicators Construction-Zhou Limin | The National Bureau of Statistics Website | Avavailable directly |
| Urbanization rate *C_3_* | The National Bureau of Statistics Website | The National Bureau of Statistics Website | Urban Population / Total Population |
| Density of water supply pipeline in built-up area *C_14_* | China Urban Construction Statistics Yearbook | China Urban Construction Statistics Yearbook | Avavailable directly |
| GDP per capita *C_21_* | Study on China Urban Disaster Resilibility Assessment and Its Improvement Strategy-Li Ya | China Urban Statistical Yearbook | Direct access |
| Per capita disposable income of urban residents *C_22_* | The National Bureau of Statistics Website | The National Bureau of Statistics Website | Direct access |
| Water resources per capita *C_4_* | Study on Elastic Capacity Assessment of Urban Water Supply System under the Impact of Salt Tide Situation-Liu Jian | The Provincial Water Resources Gazette | Direct available |
| Water consumption per 10,000 RMB of industrial added value *C_5_* | The National Bureau of Statistics Website | The National Bureau of Statistics Website | Industrial water consumption / industrial added value |
| Urban sewage treatment rate *C_10_* | China Urban Construction Statistics Yearbook | China Urban Construction Statistics Yearbook | The (%) is directly available |
| Water consumption per 10,000 RMB GDP *C_6_* | The National Bureau of Statistics Website | The National Bureau of Statistics Website | Total water use for all industries in the region * 10,000 / GDP |
| Investment in waste water treatment project has been completed *C_12_* | The National Bureau of Statistics Website | The National Bureau of Statistics Website | Direct access |
| Investment in fixed assets of water conservancy, environment and public facilities management industry *C_15_* | The National Bureau of Statistics Website | The National Bureau of Statistics Website | Direct access (excluding farmers) |
| Total water supply *C_11_* | The National Bureau of Statistics Website | The National Bureau of Statistics Website | Direct access |
| More old population dependency ratio *C_23_* | The National Bureau of Statistics Website | The National Bureau of Statistics Website | The (%) is directly available |
| Economize water consumption *C_26_* | China Urban Construction Statistics Yearbook | China Urban Construction Statistics Yearbook | Available directly |
| Water consumption exceeding the planned quota *C_27_* | China Urban Construction Statistics Yearbook | China Urban Construction Statistics Yearbook | Available directly |
| Natural population growth rate *C_25_* | Study on China Urban Disaster Resilibility Assessment and Its Improvement Strategy-Li Ya | China Urban Statistical Yearbook | The (‰) is directly available |
| Urban registered unemployment rate *C_24_* | Toughness City: Risk Management and Indicators Construction-Zhou Limin | The National Bureau of Statistics Website | The (%) is directly available |
